# Co‐Creating an Interconception Infographic for Women From Priority Populations for Inclusion in the Baby Bundle at a Victorian Hospital: A Feasibility and Acceptability Pilot Study

**DOI:** 10.1111/ajo.70154

**Published:** 2026-06-10

**Authors:** Asvini K. Subasinghe, Joyce Xiao, Shruthi Patlori, Tyla Parker, Nuhansi Wijesinghe, Sally Cussen, Penny Sheehan, Jacqueline A. Boyle

**Affiliations:** ^1^ Department of Health Systems and Equity Eastern Health Clinical School, Monash University Melbourne Australia; ^2^ Eastern Health, Box Hill Hospital Melbourne Australia

## Abstract

Providing high‐quality reproductive health information for pregnant women will promote health literacy, improve postpartum maternal health and optimise preconception health before future pregnancies. We analysed antenatal data from 314 women attending a Victorian Hospital for pregnancy services, and co‐designed and evaluated a culturally responsive interconception health tool for inclusion in the baby bundle provided to women attending this service. Information around family planning, nutrition and physical activity, lactation, common conditions in pregnancy, mental health and support networks was included in the tool. Women reported that combining an interconception resource with the standard midwifery‐led discharge consultation would be optimal.

## 1 Introduction

The interconception period, defined as the time between pregnancies, is increasingly recognised as a pivotal opportunity to optimise maternal health, reduce the likelihood of adverse outcomes in subsequent pregnancies, and address unresolved reproductive health needs such as contraceptive behaviours and managing chronic conditions such as endometriosis [1, 2]. Interconception care encompasses medical, psychological, behavioural and/or interventions to address modifiable risk factors that may adversely affect future pregnancies and long‐term health [1]. However, its integration into routine maternity services remains inconsistent and inequitable, particularly impacting maternal and child health outcomes for women from culturally and linguistically diverse (CALD) backgrounds [3].

In Australia, women from CALD backgrounds face inequitable access to interconception care [3]. Barriers such as poor health literacy, limited culturally appropriate resources, cost, and time constraints during clinical consultations reduce opportunities for effective reproductive life planning [3, 4]. Women from Sri Lankan, Indian, and Chinese backgrounds, a substantial proportion of the birthing population in Victoria, are at greater risk of poor reproductive health outcomes, including increased rates of gestational diabetes, unplanned pregnancies, and inadequate contraceptive use [5, 6]. Despite the availability of internationally developed interconception care tools, these have not been designed for use among CALD populations [7]. Findings from a recent systematic review showed that while patients expressed interest in receiving interconception health counselling, clinicians reported they experienced competing priorities, lack of interconception health knowledge, and a lack of ownership in the delivery of this information—all of which pose a significant barrier to the uptake of inclusive interconception care for all women, especially those from CALD backgrounds [8, 9]. There is also strong evidence to support that tools that are co‐designed with women, rather than for them, are better positioned to meet cultural, linguistic, and contextual needs [10, 11, 12]. The aim of our study was to co‐design a culturally responsive interconception health infographic for inclusion in the baby bundle at a Victorian hospital, a level 5 maternity service. This hospital was selected because it has a high proportion of women from CALD backgrounds attending the service.

## 2 Methods

### 2.1 Ethics Statement

This study received approval from the Eastern Health Human Research Ethics Committee (HREC) via the National Health and Medical Research Council's (NHMRC) Ethics Review Manager system (ERM). Approval was granted under protocol version 6.0 (20 August 2024). All procedures were conducted in accordance with the NHMRC National Statement on Ethical Conduct in Human Research (2007, updated 2018). Written informed consent was obtained from all participants.

### 2.2 Phase 1: Birthing Outcomes System (BOS) Data Analysis

De‐identified antenatal data were extracted from 314 randomly selected participants from the Birthing Outcomes System (BOS) at a Victorian hospital. The de‐identified data were subsequently analysed with STATA SE 18 to identify trends and needs. We used a preconception health core indicator template developed by the Preconception Health Network of Australia [13, 14]. These data were used to inform the co‐design of the infographic to ensure specific health parameters attributable to South Asian and East Asian women presenting at the hospital were included in the intervention.

### 2.3 Phase 2: Co‐Design of the Intervention

We conducted an online workshop via Zoom with 6 consumers from Sri Lankan (*N* = 1), Indian (*N* = 1) and Chinese (*N* = 4) backgrounds and individual interviews with 4 health professionals to gather feedback on culturally appropriate content to be included in the infographic. Data were audio‐recorded and transcribed verbatim. Content analysis was conducted to identify key themes by two researchers. We provided $40 gift vouchers to consumers for each hour of their time.

### 2.4 Phase 3: Pilot Feasibility Trial of the Intervention

#### 2.4.1 Intervention

The control group (*n* = 8) continued receiving their routine discharge appointment, while the intervention group (*n* = 8) was introduced to the interconception intervention as an adjunct to their routine discharge by one of the study investigators. The intervention was delivered in a 60‐minute Zoom session, which included going through the infographic and allowed participants to ask questions of the investigator. Following the end of the online session, each participant in the Intervention group received a hard copy of the infographic and booklet via mail.

#### 2.4.2 Recruitment and Consent

Eligible participants were English‐speaking women over 18 years of age from Sri Lankan, Indian, or Chinese backgrounds. Recruitment was conducted during routine antenatal visits by midwives and/or obstetricians. Women expressing interest in the study were referred to a research assistant, who provided detailed verbal and written information about the study.

Participants provided informed consent by returning signed consent forms to the research assistants either as a hard copy given in person or an electronic version sent by email.

#### 2.4.3 Randomisation

Once consented, participants were randomised using block randomisation and alternating block sizes to avoid predictable assignments in StataSE18 statistical software, by the lead study investigator, into either the intervention or control group. Each participant was assigned a unique study ID with the prefix “I” (intervention) or “C” (control).

#### 2.4.4 Evaluation

At the conclusion of the study, all participants in each group completed an identical short online knowledge, attitudes, and practices survey via Qualtrics, focused on which topics were covered in their appointment at discharge/online interconception health information session. The survey also captured feedback on participant satisfaction with the intervention and their likelihood of recommending it to others. Completed responses were transcribed by one of the co‐authors for analysis. All participants received a $40 gift card for their involvement in the study.

## 3 Results

### 3.1 Phase 1: BOS Antenatal Data

There were 4 041 records available in BOS from the period 1/4/2024 to 30/4/2024. We randomly selected ~10% of the most complete data available from this period of time, which resulted in 312 records being included for analysis in this study. The mean age of participants was 32.4 years (SD 4.3), and the median BMI was 24.9 (Q1–Q3: 22.2–29.4, Table 1). Approximately 52% were married, 18.5% were in de facto relationships, and 13.4% were single. Approximately 20% of women were born in South Asia and China.

**TABLE 1:** Descriptive analysis of socio‐demographic and preconception health indicator data for 314 women attending Box Hill Maternity Services, Victoria.

Variable	*N* (%)
Age, years Mean (SD)	32.4 (4.3)
Marital Status	
Married	164 (52.2)
Defacto	58 (18.5)
Single	42 (13.4)
Unknown	49 (15.6)
Separated	1 (1)
Employment status	
Midwife/Nurse	27 (9)
Education	21 (7)
Administration	23 (7)
Health Professional	16 (5)
Hospitality	16 (5)
IT	10 (3)
Business	12 (4)
Retail	11 (4)
Other	31 (10)
Unemployed	36 (12)
No response	105 (34)
Indigenous status, *N* = 312	
Non‐Indigenous	308 (98)
Indigenous	4 (1)
Country of Birth	
Australia	166 (53)
China	29 (9)
India	14 (4)
Malaysia	8 (3)
Burma	7 (2)
New Zealand	6 (2)
Phillipines	5 (1.6)
Pakistan	4 (1.3)
Not stated	43 (14)
Other	32 (10.2)
Ethnicity	
Chinese	35 (11)
European	16 (5)
Indian/Pakistani	23 (7)
Middle Eastern	2 (1)
Other	24 (68)
Year of arrival, *N* = 139	
1988–2008	42 (30)
2009–2024	97 (70)
BMI, Median (Q1–Q3)	24.9 (22.2–29.4)
Parity	
0	150 (48)
1	115 (37)
2	43 (14)
≥ 3	5 (1)
Past birth outcomes	
Abortion induced	25 (8)
Abortion spontaneous	28 (9)
Live birth	169 (54)
Neonatal birth	1 (0)
Still birth	2 (1)
Null	89 (28)
First antenatal visit (gestation in weeks)	
0–10	148 (47)
10–20	148 (47)
21–36	18 (6)
Antenatal COVID‐19 vaccine, *N* = 149	
Yes, before and during pregnancy	4 (2.7)
Yes preconception only	127 (85.2)
No	9 (6)
Declined to say	9 (6)
Antenatal influenza vaccine, *N* = 240	
Yes	202 (84)
No	26 (11)
Declined to say	8 (3)
Family history of diabetes	
Yes	97 (31)
No	217 (69)
Family history of hypertension	
Yes	98 (31)
No	216 (69)
Family history of mental illness	
Yes	23 (7)
No	291 (93)
History of Gestational Diabetes	19 (6)
History of PPH	26 (8)
History of Anxiety/Depression	18 (6)
Current Diabetes	
Gestational Diabetes	66 (21)
Type 1	2 (1)
Type 2	2 (1)
Pre‐eclampsia	10 (3)
Iron Deficiency/Anaemia	40 (12.7)
Advanced age pregnancy (≥ 35 years)	33 (10.5)
Substance use	
Amphetamines/cannabis/smoking ceased during pregnancy	1 (0.5)
Smoking ceased during pregnancy	7 (2)
Smoking current	5 (1)
None	301 (96)
Drinking before 20 weeks or after 20 weeks	0
Vaping before 20 weeks or after 20 weeks	0
Smoking before 20 weeks	
No	301 (96)
Yes	6 (2)
Quit	7 (2)
Smoking after 20 weeks	
No	311 (99)
Yes	2 (0.5)
Not stated	1 (0.5)
Edinburgh postnatal depression scale, *N* = 219	
0–9	196 (89.5)
10–12	14 (6.4)
13+	9 (4.1)

Nearly half (47%) had their first antenatal visit within 10 weeks of gestation, indicating timely primary care access. Around 70% of women completed the Edinburgh Postnatal Depression Scale antenatally, with 4.1% scoring high risk (13+).

Antenatal supplement use was high, with 89% (281/314) of women taking supplements, and around 25% also taking vitamin D and iron. Approximately 13% of women had iron deficiency anaemia. Gestational diabetes mellitus was diagnosed in 21% (66/314), while 1% had pre‐existing Type 1 or Type 2 diabetes (2/314).

### 3.2 Phase 2: Co‐Design Workshop and Interviews

**FIGURE 1:**
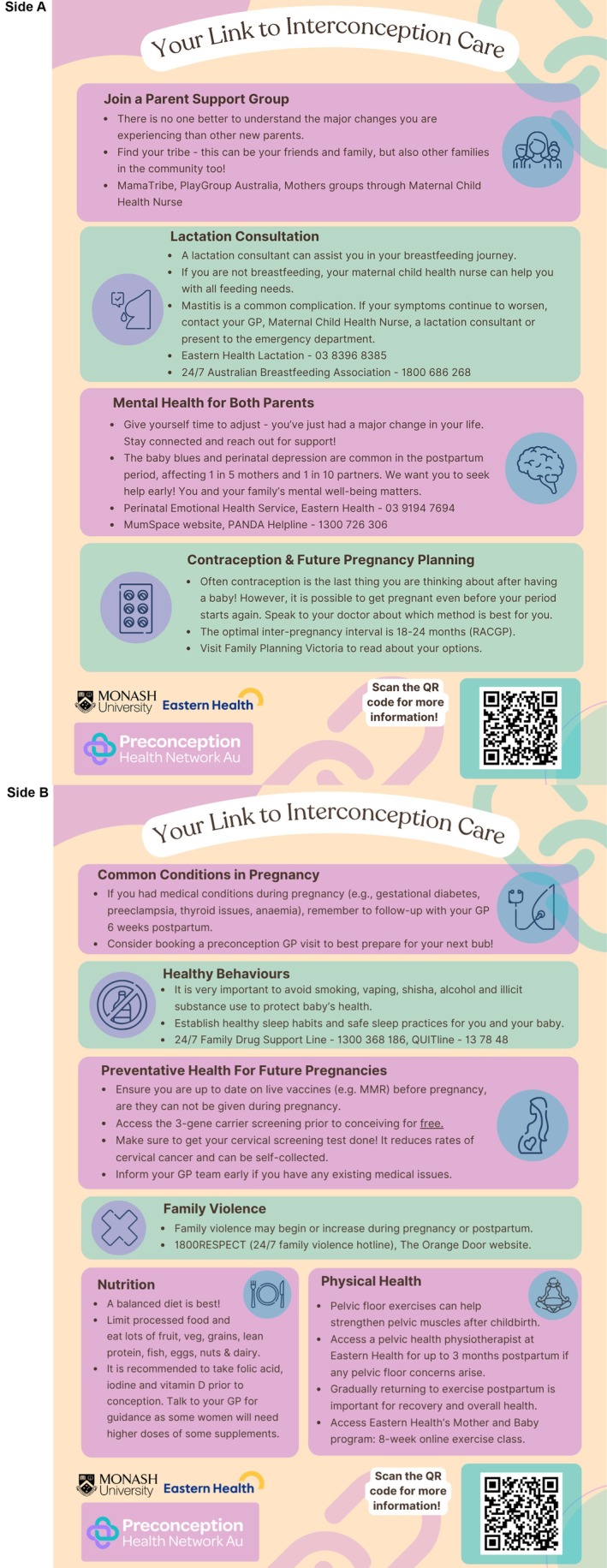
Co‐designed culturally responsive interconception infographic for inclusion in the baby bundle at a Victorian hospital.

Key content areas identified during the co‐design workshop and interviews with health care professionals were as follows: Contraception and family planning, how to find peer‐support/parent groups and lactation consultants, preventative health measures, mental health, family violence, nutrition and physical health (particularly pelvic floor exercises), and common conditions in pregnancy (Figure 1). Given the breadth of content requested to be included in the infographic, as well as common pregnancy conditions identified in antenatal data (e.g., gestational diabetes and anaemia), we decided to develop a supplementary booklet that provided further information on each of the content areas if women were interested in learning further information (Supplementary File S1).

### 3.3 Phase 3: Feasibility and Acceptability Pilot Study

A total of 16 pregnant women were recruited from the antenatal clinic at the hospital and randomised into equal groups of Intervention (*N* = 8) and Control (*N* = 8).

Participants in the Intervention group said the infographic that we developed was incredibly useful and covered additional resources in great detail:

[I.1] “I am facing a very stressful situation at the moment without family support, so good to know that there is an emotional support program that can help*”*.

Information regarding “Mothers' social and info‐sharing occasions” was also appreciated.

Participants in the Control group said they would have liked additional resources about interconception health during their visit in the form of one‐on‐one counselling (75%), digital media (75%) and printed brochures (50%). No Control participants received information *about lifestyle factors or emotional and psychological support*, but valued receiving information from their clinician about contraception and having the opportunity to ask questions. All participants said they would appreciate having an interconception infographic included in their baby bundle.

## 4 Discussion and Conclusion

We have co‐designed an interconception infographic that women in our study reported would be an excellent resource to which midwives can refer during their discharge consultation with their patient. We have also shown that the incidence of pregnancy‐associated complications such as gestational diabetes, anaemia and perinatal depression remains high in this catchment, which highlights the importance of continuing to monitor women from priority groups in the interconception period. Targeted efforts are needed to enhance preconception/interconception health counselling in areas such as preconception and antenatal supplementation, mental health screening, pre‐existing condition management, contraception, and social support.

Women of South and East Asian backgrounds in Australia experience disproportionately higher rates of pregnancy‐related complications. GDM is up to three to five times more prevalent than in women born in Australia, contributing to increased perinatal and long‐term metabolic risk [15, 16]. Anaemia in pregnancy is also more prevalent among South Asian women, influenced by nutritional and physiological factors [17]. Moreover, migrant Asian women experience greater rates of symptoms of perinatal depression due to cultural stigma, limited support and migration‐related stressors [18]. Together, these culturally specific risk profiles highlight the need for culturally responsive and appropriate interconception care involving targeted health education resources.

This is a pilot study and therefore limitations included a small sample size and largely descriptive outcome measures. Additionally, the women received an hour‐long discussion, which may not be feasible in routine care. In future, we aim to trial this infographic in a larger sample across the pilot site and subsequently multiple hospitals in Australia and explore ways to implement the infographic within the context of limited time and staff. For example, this could include additional Supporting Information, such as accompanying videos from health experts.

We have successfully co‐developed, implemented and evaluated a culturally responsive interconception infographic and booklet for inclusion in the baby bundle at a Victorian Hospital. This resource is also now available on the hospital's digital health platform, with evaluation planned. Implementation and evaluation at a larger scale could be adapted in other pregnancy care services across Australia.

## Funding

This work was supported by Eastern Health Foundation.

## Conflicts of Interest

A.K.S., J.X., P.S. and J.B. designed and conceived the study. S.C., T.P. and N.W. were responsible for data collection. A.K.S. and T.P. drafted the manuscript; A.K.S., S.P. and N.W. analysed data. All authors provided a final review of the manuscript.

## Supporting information


**File S1:** Interconception Health Information Booklet.

## Data Availability

The data that support the findings of this study are available on request from the corresponding author. The data are not publicly available due to privacy or ethical restrictions.
