# Supplementary material for: Co‐Creating an Interconception Infographic for Women From Priority Populations for Inclusion in the Baby Bundle at a Victorian Hospital: A Feasibility and Acceptability Pilot Study
Source: Aust N Z J Obstet Gynaecol. 2026 Jun 10;66(3):e70154. doi: 10.1111/ajo.70154 (PMC13254225; doi:10.1111/ajo.70154)
Supplement: Supplementary file 1 — File S1: Interconception Health Information Booklet. [file AJO-66-0-s001.pdf]

Eastern Health

MONASH University

Preconception Health Network Au

Your Link to Interconception Care

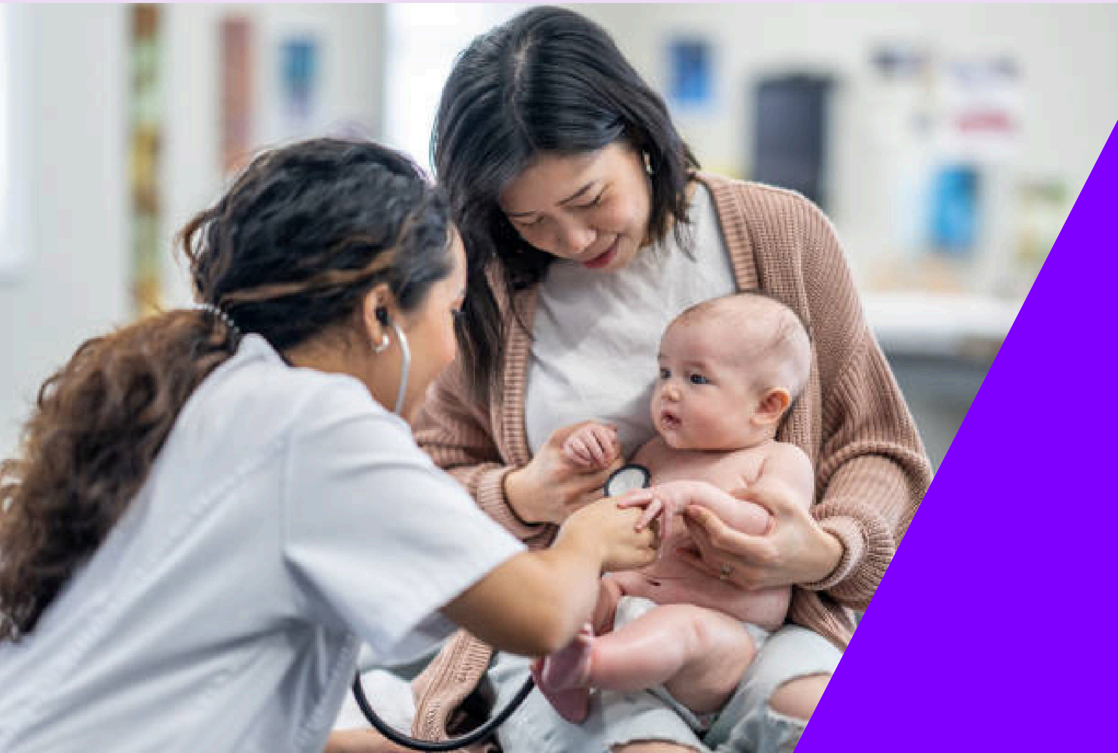

Contents

→ Join a Parent Support Group

→ Family Violence

→ Lactation Consulatation

→ Common conditions in pregnancy

→ Mental Health for Both Parents

→ Preventative Health For Future Pregnancies

→ Contraception and future pregnancy planning

→ Nutrition

→ Healthy behaviours

→ Physical Health

Join a Parent Support Group

- Parent-child support groups offer a welcoming space to connect with other parents, share experiences, and gain valuable advice on parenting challenges. These groups provide emotional support, enhance parenting skills, and promote child development through play and social interaction. Attending helps build a strong support network, reduces feelings of isolation, and boosts your confidence as a parent. Join a support group to nurture both your well-being and your child's growth in a supportive community.
- **Resources:** MamaTribe, PlayGroup Australia, Mothers groups through Maternal Child Health Nurse

Lactation Consultation

- A lactation consultant provides expert support for breastfeeding, helping you navigate common challenges such as latching difficulties, low milk supply, or nipple pain. They offer personalised advice to ensure your baby is feeding well and thriving, while also supporting your comfort and confidence. Accessing lactation support early can improve breastfeeding success, enhance bonding with your baby, and promote better health outcomes for both mother and child.
- **Resources:** Visit 'Lactation Support Unit' on the Eastern Health website, 24/7 Australian Breastfeeding Association - 1800 686 268
- Maternal child health nursing services can also assist in arranging these visits

Mental Health For Both Parents

- Taking care of your mental health is essential for your well-being and your family's. Managing stress, seeking support, and practicing self-care can improve your mood and energy. Stay connected with loved ones, engage in activities you enjoy, and don't hesitate to reach out to a healthcare provider or counsellor if you need help. A healthy mind helps you navigate parenthood with confidence and fosters a positive environment for your child's development.
- Planning for a baby, pregnancy and postpartum are all periods of time in a woman's life that involve large amounts of change. Your healthcare providers will likely ask you about your current and past emotional and social wellbeing, to make sure they can best help you through this difficult time.
- The baby blues and perinatal depression are common in the postpartum period, affecting 1 in 5 mothers and 1 in 10 partners. We want you to seek help early! You and your family's mental well-being matters.
- **Resources:** Perinatal Emotional Health Service (PEHS) at Eastern Health - (03) 9194 7694, MumSpace website, PANDA Helpline - 1300 726 306

Contraception and Future Pregnancy Planning

- Contraception gives both women and men the ability to plan for the right time to have children, with a variety of options available to suit different needs. Whether you're choosing hormonal, barrier, or long-term methods, contraception helps space pregnancies and ensures optimal health between births, allowing you time to recover and prepare for the next step in your family's journey.

	Lactational Amenorrhoea	Condoms	Mini Pill	Implanon	Mirena IUD	Tubal Ligation	Vasectomy
When implemented	Immediately, if breastfeeding	Immediately	Immediately	Immediately after birth	6 weeks or at time of C-section	At C-section	Personal choice
Efficacy	98% for 6mo post-partum<br><br>Subject to frequency and regularity of feeding	76-99%<br><br>Subject to user error	93-99%<br><br>Works well if used on time	>99%<br><br>Set & forget	>99%<br><br>Set & forget	>99%<br><br>Permanent	>99%<br><br>Permanent
Side-effects	Vaginal dryness, reduced libido	Minimal, allergy	Headaches, nausea, breast tenderness	Spotting, irregular bleeding, headaches, nausea	Spotting, irregular bleeding, headaches, nausea	Bleeding, infection	Bleeding, infection

*Lactational amenorrhea: Only lasts for up to 6mo postpartum, while amenorrhoeic and fully breastfeeding. Fully breastfeeding includes feeding day and night, no feeding supplementation, and no long intervals between feeds (>4hrly during day or 6hrly at night).
https://www.acog.org/womens-health/faqs/postpartum-birth-control

Healthy Behaviours

- Maintaining healthy behaviours is crucial when caring for a newborn and when preparing for your next pregnancy.
- Avoid smoking, vaping, shisha, alcohol, and illicit substance use, as they can harm your baby's development, both in the womb and in their first crucial years of life.
- Sleepless nights can be common after having a baby! Working to establish good sleep habits is important for both you as sleep is essential for your recovery. For baby, it helps their growth and development. Employ safe sleeping arrangements for your baby, with your baby sleeping in their own space (such as a cot or bassinet that meets current Australian standards). Although co-sleeping with your baby can be more risky, if you decide sleeping is best for your family, make sure to follow tip of safe co-sleeping.
- **Resources:** 24/7 Family Drug Support Line - 1300 368 186, QUITline - 13 78 48, Red Nose Australia's 'Safe Sleeping Practices" webpage, Preganacy, Birth and Baby 'Safe sleep for babies' webpage.

Family Violence

- Pregnancy and postpartum is a vulnerable time for many families, and unfortunately, family violence can start or worsen during this period.
- Family violence can take many forms, including physical, sexual, emotional, psychological, financial, verbal abuse and social isolation.
- If you feel unsafe or threatened, it's vital to seek support! Speak to any healthcare professional, social worker, or contact domestic violence helplines and services in your area. You and your baby deserve to live in a healthy, supportive environment, and help is always available.
- **Resources:** 1800RESPECT 24/7 family violence hotline (1800 737 732), Lifeline for crisis support (13 11 14), The Orange Door website, White Ribbon Australia website.

Common Conditions in Pregnancy

- If you had medical conditions during pregnancy, remember to follow-up with your GP 6 weeks postpartum.
- Some prominent conditions during pregnancy are gestational diabetes, gestational hypertension, thyroid issues, anaemia and hypothyroidism.
- Gestational diabetes (high blood sugar during pregnancy) requires careful management to reduce risks like a large baby or neonatal hypoglycaemia. Gestational hypertension (high blood pressure during pregnancy) may need medication as it can lead to complications like preeclampsia or eclampsia. Anaemia, particularly iron deficiency, can cause fatigue, breathlessness, and impact baby's growth, often requiring iron supplements.
- Hypothyroidism, when the thyroid doesn't produce enough hormones, may require medication and dietary changes.
- Additionally, optimising pre-existing medical conditions before conception is crucial. Consult your doctor to manage your ongoing health issues, promoting the best outcomes for you and your baby.
- **Resources:** Pregnancy Birth and Baby 'Complications during pregnancy' webpage.

Preventative Health for Future Pregnancies

- Before your next pregnancy, it's important to focus on preventative health for the best outcomes.
- Make sure you're up to date with live vaccines like MMR, as these can't be given during pregnancy. During pregnancy, free vaccines for influenza, whooping cough, and RSV are available to protect both you and your baby.
- Discuss your medical history with your healthcare team, including blood disorders or being RhD negative, as these may need extra screening.
- Consider genetic testing like the 3-gene carrier screen or NIPT to assess risks.
- Lastly, schedule your cervical screening to detect HPV and lower cervical cancer risk.
- **Resources:** Better Health Channel 'Immunisation and Pregnancy' webpage, Centre for Genetics Education 'Screening tests during pregnancy' webpage, Cancer Council 'A guide to: Cervical cancer screening' webpage.

Nutrition

- Maintaining a balanced diet is crucial for both preconception and pregnancy. Focus on vegetables, fruits, whole grains, lean meats, fish, eggs, nuts, and dairy while limiting processed foods.
- It is recommended to take 0.5mg of folic acid daily for at least one month before conception and during the first three months to reduce the risk of brain and spinal cord defects. Iodine (150mcg daily) should be taken throughout preconception and pregnancy to support thyroid function and brain development.
- While pregnancy multivitamins are optional, avoid those with doses higher than the recommended daily intake.
- Increase fiber intake to reduce pregnancy-related constipation.
- Always consult your health practitioner for personalised advice.
- **Resources:** Australian government fact sheet 'Nutrition advice during pregnancy', Eat for Health 'Healthy eating during your pregnancy' webpage, Better Health Channel 'Pregnancy and diet' webpage.

Physical Health

- Physical activity is key to postpartum recovery and overall health.
- Pelvic floor exercises are particularly important for strengthening pelvic muscles after childbirth and preventing issues like incontinence. If you experience any pelvic floor concerns, consider accessing a pelvic health physiotherapist at Eastern Health for support up to 3 months postpartum.
- Gradually returning to exercise helps with recovery, improves mood, and boosts energy levels.
- **Resources:** Pregnancy Birth and Baby 'Pelvic Floor Exercises' webpage, Continence Health Australia 'Pelvic Floor Exercises for Women' webpage, Pregnancy Birth and Baby 'Safe return to exercise after pregnancy' webpage.
- Eastern Health offers an 8-week online educational exercise class through its Mother and Baby program, which is designed to support new parents in regaining strength and mobility. This program costs $10 per session and provides valuable guidance to help you return to physical activity safely and effectively.

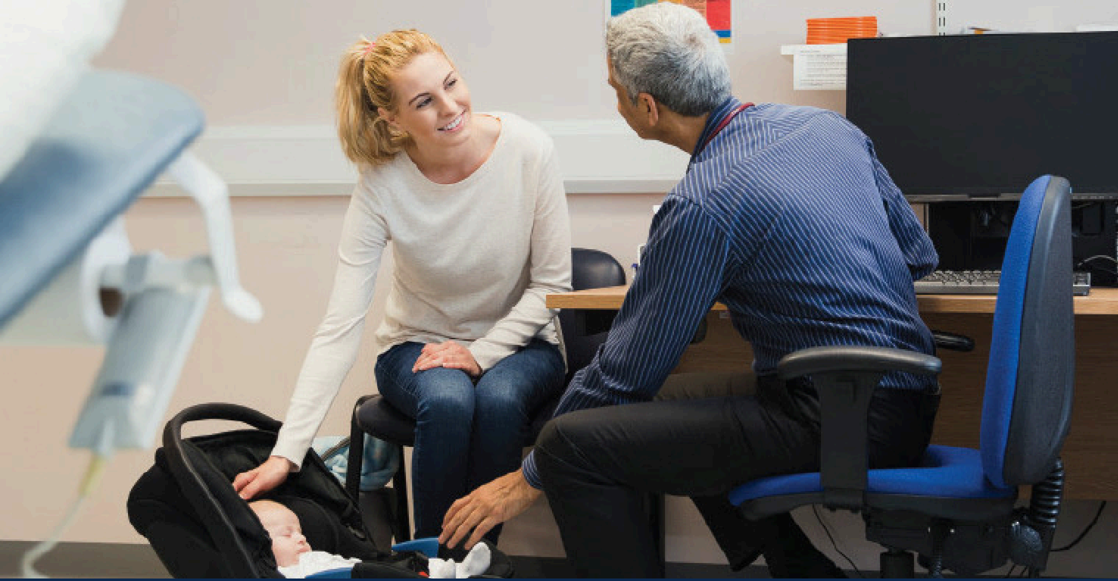

Further Reading

- Eastern Health Maternity Services - https://www.easternhealth.org.au/services/maternity-services/
- Eve App - found on Apple App Store and Google Play Store
- Women's mental health - https://womensmentalhealth.org/specialty-clinics/postpartum-psychiatric-disorders-2/
- Sexual Health Victoria - https://shvic.org.au/
- Post-partum contraception - https://www.acog.org/womens-health/faqs/postpartum-birth-control
- Lactational amenorrhea, Nature article - Link
- Pregnancy Birth and Baby - https://www.pregnancybirthbaby.org.au/
- Better Health Channel - https://www.betterhealth.vic.gov.au/
- Red Nose Australia - https://rednose.org.au/section/safe-sleeping
- The Orange Door - https://www.orangedoor.vic.gov.au/
- White Ribbon Australia - https://whiteribbon.org.au/
- National Cervical Screening Program - https://www.health.gov.au/our-work/national-cervical-screening-program
- Continence Health Australia - https://www.continence.org.au/who-it-affects/women/female-pelvic-floor-muscles
- Eastern Health Mother and Baby Program - https://www.easternhealth.org.au/service/mother-and-baby-program/
